# The Relation between Concrete, Mortar and Paste Scale Early Age Properties

**DOI:** 10.3390/ma14061569

**Published:** 2021-03-23

**Authors:** Martin Klun, Vlatko Bosiljkov, Violeta Bokan-Bosiljkov

**Affiliations:** Faculty of Civil and Geodetic Engineering, University of Ljubljana, Jamova 2, SI-1000 Ljubljana, Slovenia; martin.klun@fgg.uni-lj.si (M.K.); vlatko.bosiljkov@fgg.uni-lj.si (V.B.)

**Keywords:** concrete, mortar and paste scale of cement-based material, early hydration stage, microstructure development, electrical conductivity, ultrasonic method, standard tests

## Abstract

Microstructure development of concrete, mortar, and paste scale of cement-based material (CBM) during the early hydration stage has a significant impact on CBM’s physical, mechanical, and durability characteristics at the high maturity state. The research was carried out using compositions with increased autogenous shrinkage and extended early age period, proposed within the RRT^+^ programme of the COST Action TU1404. The electrical conductivity method, used to follow the solidification process of CBM, is capable of determining the initial and final setting time, and the end of the solidification process acceleration stage for the paste and mortar scale. Simultaneous ultrasonic P- and S-wave transmission measurements revealed that the ratio of velocities *V_P_*/*V_S_* is highly dependent on the presence of aggregates—it is considerably higher for the paste scale compared to the mortar and concrete scale. The deviation from the otherwise roughly constant ratio *V_P_*/*V_S_* for each scale may indicate cracks in the material. The non-linear correlation between the dynamic and static elastic moduli valid over the three scales was confirmed. Additionally, it was found that the static E-modulus correlates very well with the square of the *V_S_* and that the *V_S_* is highly correlated to the cube compressive strength—but a separate trendline exists for each CBM scale.

## 1. Introduction

Formation and development of cement-based materials’ (CBMs) internal structure is a complex process that depends on various influencing parameters—such as the type of cement, aggregate, water-to-cement ratio, presence of chemical admixtures, and/or mineral additives, temperature, curing conditions, etc. Immediately after mixing the CBM components into a solid suspension, the cement hydration process starts. During the process, many chemical and physical changes occur that result first in the change of the CBM’s physical state (transition from a quasi-fluid state to a solid-state—setting). After the solid-state is reached, ongoing hydration reactions result in a high state of CBM’s maturity that is reflected in the low rate at which the material’s strength and elastic properties are changing with time. Upon reaching this high state of maturity, the corresponding time is known as an early hydration period that varies from several hours to a few days or even weeks, depending on the CBM’s composition and curing conditions. Understanding microstructure development in CBMs in this early hydration period is fundamental, as it has a significant impact on the physical, mechanical, and durability characteristics of CBMs. Among other mechanisms, CBMs can undergo significant volumetric changes from an early age due to an increase in temperature, moisture variations, autogenous shrinkage, etc. These volumetric changes often cause cracks inside the CBM structure or at least induce tensile stresses that reduce the tensile strength of the CBM. Such defects in the CBM can reduce the long-term safety and durability of reinforced concrete structures [1].

Standard test methods used to evaluate the physical and mechanical characteristics of the CBMs cannot provide enough in-depth knowledge of concrete behaviour during an early age and throughout its service life. There is a need for continuous monitoring of critical properties from the start of setting to the mature age of the CBMs. Many new measurement systems for early-stage testing have been developed in recent years [2]. Ultrasonic wave transmission techniques focus on determining different stages during the CBMs structure formation; they especially focus on the setting (initial and final setting times) and early age mechanical properties [3,4,5,6,7,8,9,10,11,12,13]. Acoustic emission techniques are used to detect cement particles’ settlement, segregation, cavitation, migration of water, the formation of hydrates, and early micro-cracking of CBMs in a fresh state and during the solidification process [14,15,16,17,18]. Measurement of time-dependent electrical conductivity provides information on early age mechanical properties and different stages during the CBMs structure formation; the influence of aggregate and properties of the interfacial transition zone can be estimated as well [8,19,20,21,22,23]. Nuclear magnetic resonance is used for the continuous characterization of the evolving microstructure of various cementitious materials during the setting process [24,25,26,27,28]. Among the listed measurement systems, ultrasonic techniques, in particular, have great potential to become standard testing techniques. They can be used to monitor the solidification process of the CBMs and determine elastic parameters, as well as evaluate the strength of the early age CBM due to their clear physical basis [2].

In [10,11], the process of setting and hardening was monitored using ultrasonic transversal or shear wave (S-wave) transmission velocity and dynamic elastic modulus. These two parameters have been proven more CBM setting sensitive than ultrasonic longitudinal or compression wave (P-wave) transmission velocity. The kinetics of the dynamic elastic modulus development is tightly linked with the S-wave transmission velocity development, making the perception of the hardened phase of hydration products possible. Additionally, simultaneous P- and S-wave transmission velocity measurements confirmed the correlation between the compressive or tensile strength and dynamic elastic modulus at the early age of CBMs [12]. These findings indicate that the elastic parameters are valid solidification indicators, with which the development of other mechanical characteristics of CBMs can be evaluated at an early age. Elastic material parameters are bulk density-dependent, thus containing additional and variable material parameters, essential for the mechanical properties of CBMs [13].

## 2. Research Significance

In this study, advanced non-destructive and standard destructive tests were combined to evaluate properties of CBM at three scales—the concrete scale and “identical” compositions at the mortar and paste scale. The research was carried out using materials and compositions proposed within the Extended Round Robin Testing Programme (RRT^+^) of the COST Action TU1404: Towards the next generation of standards for the service life of cement-based materials and structures [1]. The raw materials were supplied by EDF, France—connected to a broader VerCoRs programme (https://fr.xing-events.com/EDF-vercors-project.html accessed on 14 March 2021). The University of Ljubljana (UL) was one of 42 research institutions involved in the RRT^+^ and was responsible for mechanical properties testing protocols. An additional task for the research group was collecting and analysing the mechanical properties test results. Results of 15 laboratories were available for analyses, predominantly for the VerCoRs concrete composition with a water-to-cement ratio of 0.52 and specimens aged 7 and 28 days [29,30]. Modified CBM composition (water-to-cement ratio 0.40) was tested as well, but only 3 laboratories reported test results for specimens aged 7 days or less. Moreover, only UL reported results for the tensile and flexural strength and modulus of elasticity on the paste and mortar scale. The results obtained by UL showed a decrease in the tensile strength with increasing age for the paste and mortar scale of the two CBMs during the early hydration stage [31,32]. As there were no results available for comparison in the RRT^+^ programme, the raw materials still available were used for a new, more comprehensive study. It was designed and conducted to confirm or reject those observations and as proof of concept for a broader study about the properties of cement and lime-based masonry mortars.

This study aims to monitor CBM’s solidification kinetics and the development of mechanical properties at an early age from up to 7 days. The studied RRT^+^ cement paste, mortar and concrete scale compositions are those with a water-to-cement ratio of 0.4 to increase the autogenous shrinkage of the CBM. The superplasticizer used delayed the setting of the CBM considerably and thus extended the length of the early age period. The electrical conductivity measurements were used to follow the solidification process of a particular CBM, from the state of solid suspension to the age of 7 days. On hardened specimens that were able to withstand handling operation and self-weight, the transmission times of ultrasonic P- and S-waves were measured simultaneously and standard destructive tests were performed to determine both dynamic and static mechanical properties. Based on the results of the performed tests, the correlations between different mechanical properties over the three CBM scales and between dynamic and static characteristics were determined and mutually compared; they were also compared to results obtained by other researchers.

## 3. Materials and Methods

### 3.1. Materials and Compositions

**Figure 1:**
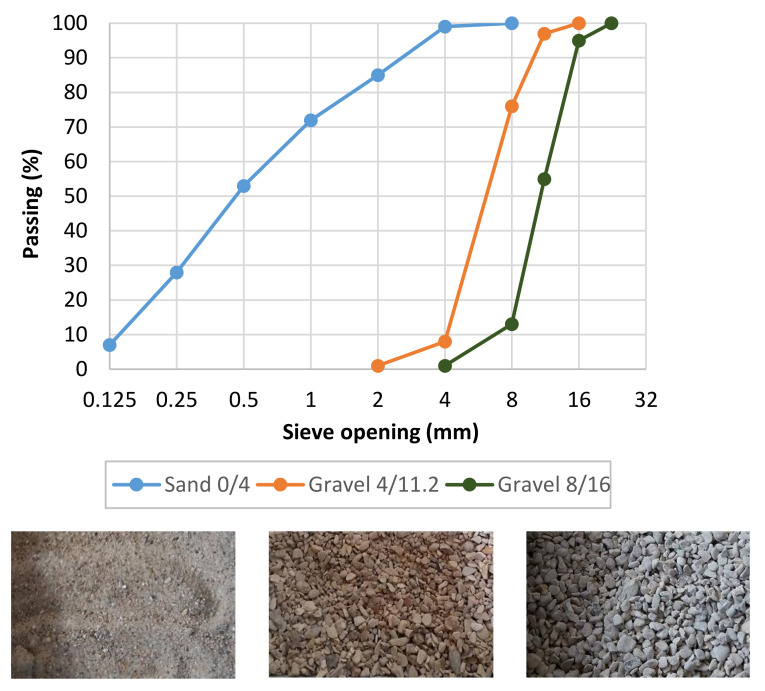
Grain size distribution and appearance of the 0/4, 4/11.2 and 8/16 fractions.

The cement used was CEM I 52.5 N CE CP2 NF Gaurain (CCB, Gaurain, Belgium). The cement’s Blain specific surface area was 4400 cm^2^/g, and its density was 3.19 g/cm^3^. The chemical composition of the cement is given in Table 1. The fine aggregate used was predominantly siliceous (72% of SiO_2_ and 13% of CaCO_3_) sand of 0–4 mm fraction, with a density of 2580 kg/m^3^ and water absorption equal to 0.77%. The coarse aggregate used was a combination of siliceous-calcareous gravel of a 4–11 fraction (R GSM LGP1) and 8–16 fraction (R Balloy). The 4–11 and 8–16 fractions density were 2530 kg/m^3^ and 2580 kg/m^3^ and water absorption were equal to 2.61% and 2.25%, respectively. The Los Angeles abrasion coefficient of the gravel fractions was less than 30. The grain size distribution and appearance of the fractions are given in Figure 1. As an admixture, the superplasticizer SIKAPLAST Techno 80 (SP) was used to obtain adequate workability of CBM mixtures.

Compositions of tested CBM scales, concrete (MOC), mortar (MOM) and cement paste (MCP) are presented in Table 2. All compositions were prepared with a water-to-cement ratio of 0.4 and a relatively high SP content to achieve a higher magnitude of autogenous shrinkage and a higher magnitude of strength at an early age. The three mixtures were “identical” mixtures on concrete, mortar, and paste scale: MOM was obtained by excluding the gravel from the MOC, and MCP was obtained with gravel and sand exclusion from the MOC. As a result, the cement content increased considerably from concrete to mortar and paste scale when compositions were given per 1 m^3^ volume (Table 2). The SP percentage was reduced for the MCP composition to provide the fresh paste mixture’s stability.

Details of materials and compositions used to prepare CBMs are available in the Testing protocols of the RRT^+^ programme [33].

It is important to note that the properties of the mortar and concrete scale of the CBM are highly related to the aggregate properties. Recent studies on CBMs have shown that, besides properties commonly reported for aggregates (such as the geological nature, density, water absorption and grain size distribution), shape regularity, shape ratio, surface texture, chemical composition, and LA abrasion resistance are additional parameters that significantly influence physical and mechanical properties of the CBM [34,35]. Therefore, the reported results are valid for the aggregates used in this study and should not be generalised.

### 3.2. Preparation of Test Specimens and Fresh Properties Testing

All the basic materials, including water, were stored under controlled laboratory conditions at a temperature of 20 ± 2 °C and relative humidity higher than 60%. The target temperature of the materials before mixing of CBMs was 20 °C. Each gravel fraction’s initial water content was measured 7 days before mixing the MOC and the water required for the fraction to reach a fully saturated surface dry state was added to the aggregate grains kept in a plastic container. After mixing of aggregate grains and added water, the container was sealed with a lid. The water content of the 4–11 and 8–16 fractions, before the mixing of MOC, was 2.76% and 2.56%, respectively and surface water was considered in calculating the water that was ultimately added to the mixer.

The mixing procedures are described in the Testing protocols of the RRT^+^ programme [33]. The mixing procedure of MCP closely followed the procedure described in standard EN 196-3 [36], and the procedure of mixing MOM and MOC followed the procedure from the standard EN 480-1 [37]. For each of the three CBM mixtures, 45 test specimens were made—9 for each testing stage. Tests were carried out in 5 stages; at the ages of 30, 36, 48, 72 h, and 7 days (168 h).

**Figure 2:**
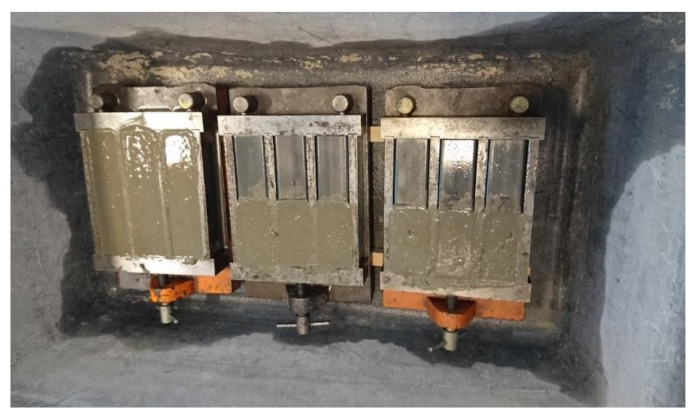
An example of a set of moulds filled with cement paste (MCP) mixture for testing at a single age.

After the mixing of MCP and MOM, the mixture temperature was measured and consistency was evaluated by flow table test according to the EN 1015-3 [38]. Due to the MCP’s high flowability, a modified procedure was applied, which excluded dropping (15-times) of the flow table. The procedure is described in [39] and the obtained value is referred to as the slump-flow value. Fresh density was evaluated following the EN 1015-6 [40] procedure. After that, mixtures were moulded into prism-shaped moulds with standard dimensions of 40 × 40 × 160 mm^3^. Three specimens were made for every single test at a particular age. For standard tests of elastic modulus, the standard prism moulds were halved to dimensions 40 × 40 × 80 mm^3^, where EN 196-1 [41] tolerance requirements were considered (Figure 2). Six specimens with dimensions of 40 × 40 × 80 mm^3^ were made for each stage of testing (3 for compressive strength test and 3 for determination of elastic modulus and *σ*-*ε* behaviour in compression).

In the case of modified ordinary concrete (MOC), the mixture temperature was measured and consistency and fresh density were evaluated following EN 12350-2 [42] and EN 12350-6 [43] procedures, respectively. Due to slump values [42] higher than 210 mm and associated reduced sensitivity of the test method [44], the spread of the specimen after the slump measurement was recorded as well. The method is similar to that used for the MCP specimen. For each testing age, three prism specimens with dimensions of 100 × 100 × 400 mm^3^, three cube specimens with dimensions of 150 × 150 × 150 mm^3^ and three cylinders with a diameter of 100 mm and height of 200 mm (100/200 mm cylinder) were produced. All the specimens in moulds were stored at relative humidity higher than 95% and a temperature of 20 ± 2 °C. Specimens for determination of elastic modulus and Poison’s ratio were equipped by double direction strain gauges, measuring longitudinal and transverse strains simultaneously.

**Figure 3:**
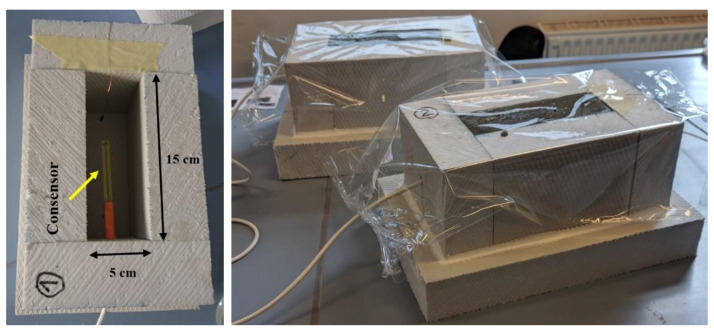
Custom-made container (**left**) and moulded specimens prepared for temperature and conductivity measurements (**right**).

Specimens for measuring temperature and electrical conductivity of mixtures were cast into the custom-made extruded polystyrene container, and the upper surface was covered with a polyvinyl sheet to avoid water evaporation, as shown in Figure 3. Inside dimensions of the container were 5 × 10 × 15 cm^3^, to ensure complete immersion of the sensors into the mixture. For each of the three mixtures, 2 parallel test specimens were prepared.

The custom-made containers were of the same dimensions as those used in [8]. In this way, the comparison of test results and determination of setting and hardening parameters of the CBMs at an early age is possible.

### 3.3. Test Methods

#### 3.3.1. Temperature and Electrical Conductivity Sensor System

Temperature and electrical conductivity in the CBM mixtures were measured with a commercial Consensor 2.0 system (Consensor B.V., Rotterdam, The Netherlands) [19], which follows the early age evolution of the CBMs properties as a function of time.

In each polystyrene container, a sensor was positioned at the specimen’s centre, as shown in Figure 3. In this way, it was completely enclosed with the CBM mixture. Continuous measurements (in 10-min intervals) of conductivity and temperature for each CBM mixture started immediately after the mixture was moulded into the polystyrene container and continued for the first 7 days of CBMs specimens’ setting and hardening. All that time, containers with specimens were stored in a laboratory under a controlled temperature of 20 ± 2 °C.

#### 3.3.2. Ultrasonic Method

**Figure 4:**
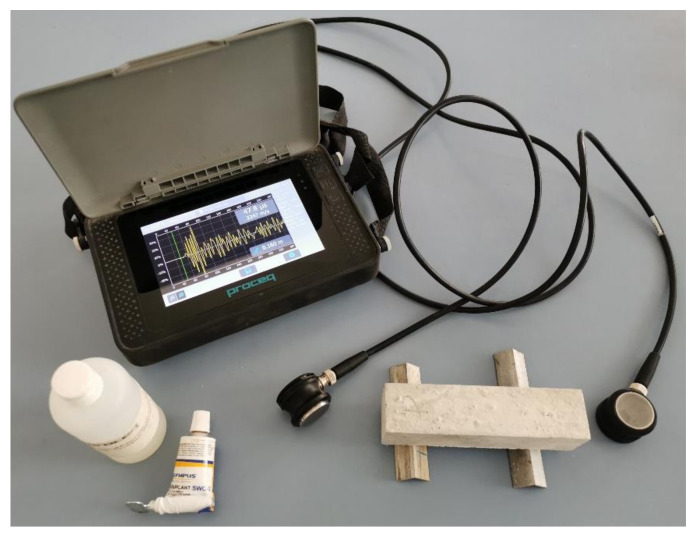
Ultrasonic device Proceq Pundit PL-200.

**Figure 5:**
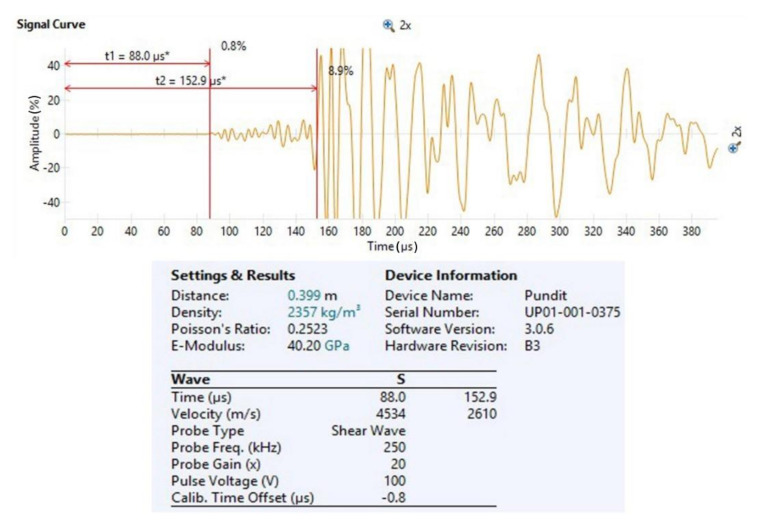
Determination of the dynamic elastic properties using the Pundit device.

For measuring the transmission time of longitudinal ultrasonic waves (P-waves) and ultrasonic shear waves (S-waves), the Proceq Pundit PL-200 device (Figure 4) (Proceq, Schwerzenbach, Switzerland) was used, with an operating frequency range of 20 kHz to 500 kHz. This range of frequencies is appropriate for the research of building materials [4]. With the ultrasonic method, the dynamic elastic modulus (*E_d_*), the dynamic Poisson’s ratio (*ν_d_*) and dynamic shear modulus (*G_d_*) were determined (Figure 5). Measurements were made on the MCP and MOM specimens with 40 × 40 × 160 mm^3^ dimensions and on MOC specimens with 100 × 100 × 400 mm^3^, using the shear waves probes with a frequency of 250 kHz. Tests were carried out before standard testing on specimens aged 30, 36, 48, 72 h and 7 days.

A single MCP, MOM, and MOC specimen was installed between transducers, converting electric voltage to ultrasonic waves and vice versa. Both transducers have to be firmly pressed on both sides of specimens to transmit ultrasonic waves directly. Complete contact between the transducer and surface of the specimen is extremely important to prevent the ultrasonic signal’s reflection and losses. For this reason, a thin layer of coupling gel was used. For transducers of ultrasonic P-waves, the coupling gel could be glycerine, but it has to be a high viscosity gel for S-waves transducers. When measuring the S-wave transmission time, the transducers’ correct settlement position has to be controlled because the S-wave signal is the strongest when transducers are parallel to each other. In the opposite case, when transducers are perpendicular to each other, the signal can disappear. In literature [5,6], the Hilbert transformation algorithm is shown to identify ultrasonic S-wave transmission. The Hilbert transformation changes the signal into an analytical shape with a defined amplitude and phase.

Ultrasonic P- and S-wave velocity within a material heavily depends on the material’s density, elastic modulus, shear modulus and Poisson’s coefficient. Before determining the elastic properties at different ages, the specimen’s density was calculated according to the standard EN 1015-10 [45]. The correlation between the mentioned parameters and ultrasonic wave velocity has been determined for isotropic homogenous materials [46]. In general, the modulus of elasticity *E* refers to the axial deformation of a material due to the P-waves, whereas the shear modulus refers to the shear deformation of a material caused by the S-waves [2,47]. The ratio between P- and S-wave transmission velocity is highly dependent on Poisson’s ratio, which presents the ratio between the axial and shear deformation [7]. The CBMs are homogenous, isotropic elastic materials; therefore, the equations for calculating dynamic elastic parameters exist. Index *d* stands for the dynamic elastic parameters, and the equations (Equations (1)–(3)) are based on the transmission velocity of ultrasonic P- and S-waves and bulk density of the material [2,46]:

(1) $$\nu_{d}=\frac{V_{P}^{2}-2V_{S}^{2}}{2\left(V_{P}^{2}-V_{S}^{2}\right)}$$

(2) $$E_{d}=V_{P}^{2}\rho \frac{\left(1+\nu_{d}\right)\left(1-2\nu_{d}\right)}{\left(1-\nu_{d}\right)}=2V_{S}^{2}\rho \left(1+\nu_{d}\right)$$

(3) $$G_{d}=\frac{E_{d}}{2+2\nu_{d}}=V_{S}^{2}\rho$$

where *ν_d_* stands for dynamic Poisson’s ratio, *V_P_* is the transmission velocity of ultrasonic P-waves, *V_S_* is the transmission velocity of ultrasonic S-waves, *E_d_* is the dynamic elastic modulus, *G_d_* the dynamic shear modulus and *ρ* is the density of CBM.

#### 3.3.3. Compressive Strength and Tensile Strength

**Figure 6:**
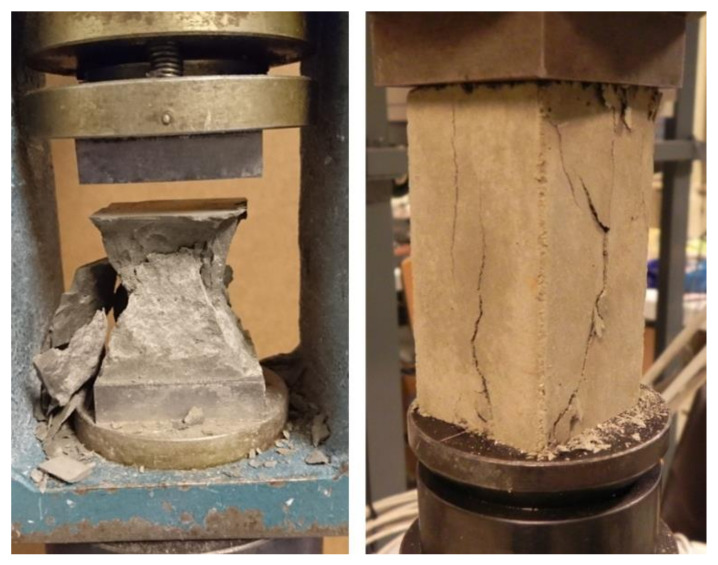
An example of MCP cube compression test (**left**) and MCP prism compression test (**right**).

The compressive strength *f_c_* for MCP and MOM specimens was assessed following the standard EN 1015-11 [48], on the halves of 40 × 40 × 160 mm^3^ prism, after the flexural test. For determination of nominal compressive stress, needed for cyclic loading/unloading protocol to determine the modulus of elasticity, the prism specimens of dimensions 40 × 40 × 80 mm^3^ were used. An example of compressive tests performed is shown in Figure 6. The compressive strength of the MOC was determined by following the standard EN 12390-3 [49], on the 150 mm cube and 100/200 mm cylinder.

Two different methods were used to evaluate the CBMs behaviour in tension: the flexural test and tensile splitting test. For determination of the flexural strength, the 3-point bending test was performed. The flexural strength of the MCP and MCM 40 × 40 × 160 mm^3^ specimen was determined by the standard EN 1015-11 [48], and in the case of the MOC 100 × 100 × 400 mm^3^ prisms, the procedure in standard EN 12390-5 [50] was followed. The tensile splitting test was carried out on the prism halves that remained after the flexural test, following the standard EN 12390-6 [51] (the modified procedure was used for the MCP and MOM specimens).

#### 3.3.4. Static Modulus of Elasticity, Poisson’s Ratio and *σ*-*ε* Behaviour in Compression

Static unloading modulus of elasticity (*E_s_*) and static Poisson’s ratio from compression (*ν_s_*) were determined by following a modified protocol [33] of standard EN 12390-13, method B [52], on 40 × 40 × 80 mm^3^ prism for the MCP and MOM, and on 100/200 mm cylinder for the MOC.

In this method, there are 3 loading cycles and the nominal stresses of each cycle, where the higher nominal stress is *σ_a_*, the lower nominal stress is *σ_b_* and the preloading stress is *σ_p_*. The preloading stress was equal to the lower nominal stress *σ_b_* in our study. With the measured compressive strength *f_c_* on the parallel specimens, the preloading and higher nominal stress were calculated using equations (Equations (4) and (5)).

(4) $$\sigma_{p}=0.1\times f_{c}$$

(5) $$\sigma_{a}=0.25\times f_{c}$$

The loading speed was 1.5 ± 0.125 MPa/s for the MCP and MOM prisms and 0.6 ± 0.2 MPa/s for the MOC composition. Static elastic modulus *E_S_* is calculated as a stress difference divided by longitudinal strain (*ε_L_*) difference that corresponds to the stress difference at the unloading of the third cycle (Equation (6)). Static shear modulus *G_S_* is calculated as a stress difference divided by the absolute value of transverse strain (*ε_T_*) difference that corresponds to the stress difference at the unloading of the third cycle (Equation (7)). Poisson’s ratio *ν_s_* is calculated as the absolute value of the transverse strain difference divided by the longitudinal strain difference (Equation (8)).

(6) $$E_{s}=\frac{\Delta \sigma }{\Delta \varepsilon_{L}}=\;\frac{\sigma_{a}-\sigma_{p}}{\varepsilon_{L}\left(\sigma_{a}\right)-\varepsilon_{L}\left(\sigma_{p}\right)}$$

(7) $$G_{s}=\frac{\Delta \sigma }{\Delta \varepsilon_{T}}=\;\frac{\sigma_{a}-\sigma_{p}}{\left|\varepsilon_{T}\left(\sigma_{a}\right)-\varepsilon_{T}\left(\sigma_{p}\right)\right|}$$

(8) $$\nu_{s}=\frac{\Delta \varepsilon_{T}}{\Delta \varepsilon_{L}}\;=\;\frac{\left|\varepsilon_{T}\left(\sigma_{a}\right)-\varepsilon_{T}\left(\sigma_{p}\right)\right|}{\varepsilon_{L}\left(\sigma_{a}\right)-\varepsilon_{L}\left(\sigma_{p}\right)}$$

**Figure 7:**
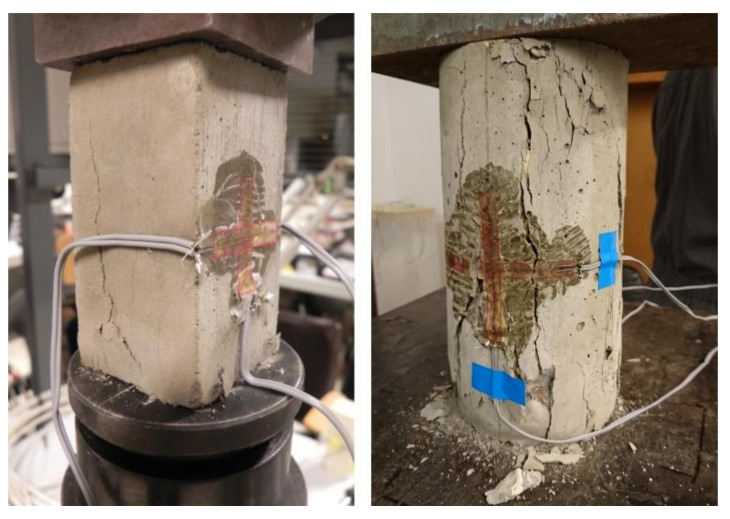
An example of a prism specimen of MCP/ mortar (MOM) (**left**) and cylinder specimen of concrete (MOC) (**right**) after the modulus of elasticity, Poisson’s ratio and *σ*-*ε* test.

After the third cycle of the test was performed, the specimen was loaded to its collapse (Figure 7). In this way, a compressive stress-strain diagram (*σ-ε* behaviour) was obtained for each specimen.

## 4. Results and Discussion

### 4.1. Fresh Properties of CBMs

After mixing each of the CBMs, the fresh properties of CBM mixtures were measured. The results are given in Table 3 for MCP and MOM and in Table 4 for MOC.

As expected, the MCP and MOM mixture temperature was very close to 20 °C. The MCP’s high slump-flow value indicated the paste’s self-compacting ability and density below 2000 kg/m^3^ was due to the exclusion of the aggregate particles. The flow value of the MOM was highly reduced due to the addition of the sand, despite the increased superplasticizer/cement ratio. However, it was still much higher than 200 mm, which was the lower limit value for the soft mortar that is cast in the bulk density measuring vessel without compaction [40]. There was no direct relationship between the MCP and MOM flow value due to the modified testing procedure applied for the MCP.

MOC fresh properties were measured for five different batches, prepared for different testing ages of the hardened concrete (Table 4). The fresh MOC temperature was in a narrow interval of 19.5 ± 1 °C, and the rest of the properties’ repeatability was high. The flow value (slump-flow) was a better indicator of the MOC consistency than the slump value. The slump value above 220 mm was not sensitive enough to evaluate a change of consistency. The MOC concrete can be classified as highly flowable concrete and not far from the self-compacting ability.

### 4.2. Time-Dependent Temperature and Electrical Conductivity Development in CBMs

**Figure 8:**
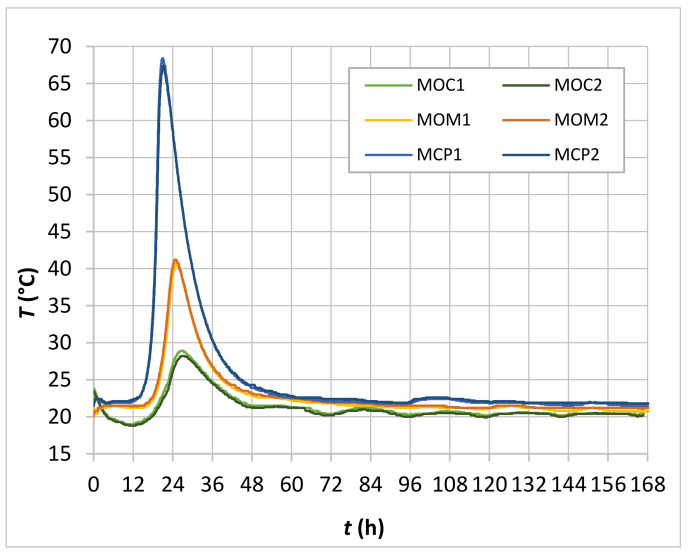
Time-dependent temperature for MCP, MOM, and MOC specimens.

The time-dependent temperatures of two MCP, two MOM and two MOC specimens within the seven-day measuring period are shown in Figure 8.

There was practically no temperature difference between the curves of the two specimens of individual CBM mixture. However, when comparing MCP, MOM, and MOC specimens’ temperature curves, it was evident that the cement content of the CBMs predominantly influenced generated heat and with it the rate of temperature increase and maximum value of the measured temperature. The custom-made extruded polystyrene container that obstructed the heat loss through five of six surfaces during the hydration process was predominantly responsible for the behaviour observed.

The development of physical and mechanical properties of CBMs was influenced by both time and temperature [53]. Due to significantly different temperature histories of the CBMs at an early age (Figure 8), the equivalent age method was used to estimate the MCP, MOM, and MOC’s time-dependent conductivity. For this purpose, the temperature influence on the effective concrete age *t_e_* was considered, using the Freiesleben-Hansen and Pedersen (Arrhenius) expression [54], with a constant activation energy of 33.500 J/mol/K determined by Leusmann et al. [55] for the CBM compositions studied.

**Figure 9:**
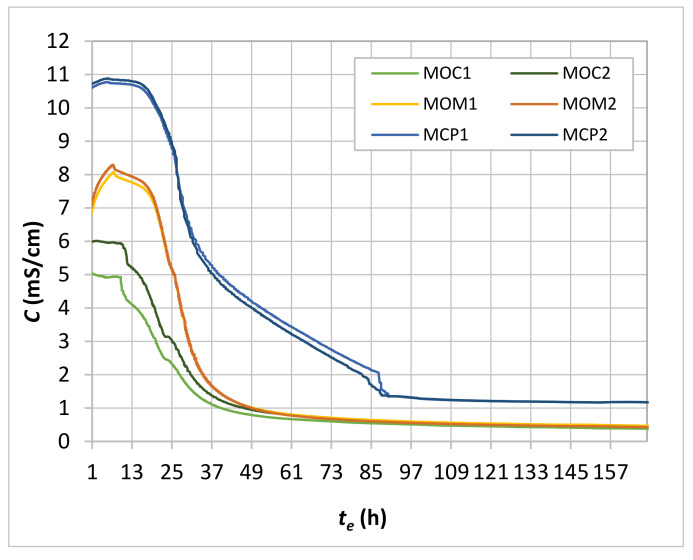
Time-dependent conductivity (*C−t_e_*) for MCP, MOM, and MOC specimens.

MCP, MOM, and MOC specimens time-dependent conductivity (*C−t* curve) measured for 7 days is shown in Figure 9. The conductivity is an indicator of the free water inside the CBM pore structure. The free water content and pore system properties depend on cement hydration level and initial water content in CBMs. Instantly after mixing, the space between cement grains (pore system) occupied by water is interconnected; at that point, the conductivity is a direct indicator of the water content in the cement paste. During the hydration process, the hydration products may interrupt the connectivity of the pore system. Consequently, the fast drop of conductivity occurs, which is reflected in the *C−t* curves’ steep decrease (Figure 9). The absolute water content of the three CBMs varied; the MCP mixture has the highest volume of water, followed by MOM and MOC mixtures. That is why the initial conductivity (before the setting process starts) was the highest in the MCP specimens and the lowest in the MOC specimens. The increase of the conductivity in the early period of hydration, observed for the MCP and MOM specimens, can be related to a dissolution of cement particles in the pore water and associated heat evolution in the first hours [20,21].

**Figure 10:**
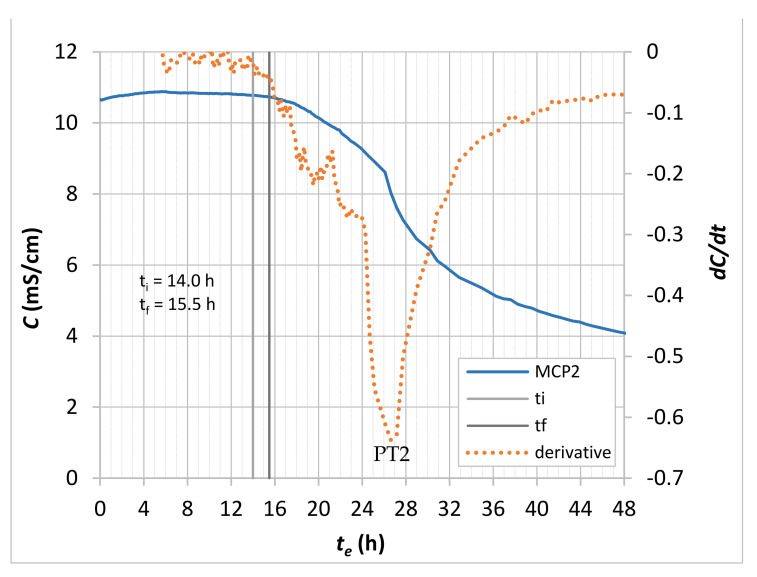
*C−t_e_* and *dC/dt* profiles of the MCP2, with the determination of initial and final setting times and PT2 time.

During the structure formation process of the CBM, several stages (initial and final setting time, percolation of solid phases, de-percolation of water-saturated pores and the time of the most intensive solidification process) are usually defined based on typical ultrasonic velocity time-dependent profiles [2]. Vogrič et al. showed [8] that a high degree of correlation exists between the P-waves ultrasonic velocity-time (*V_P_*−*t*) and electrical conductivity-time curves. By considering this correlation, the initial setting time (*t_i_*), final setting time (*t_f_*) and end of the solidification process acceleration stage (inflexion point PT2) were determined for MCP and MOM specimens (Table 5), using the electrical conductivity-time curve (*C−t*) and the rate at which conductivity changes (*dC*–*t* curve). Figure 10 illustrates how these parameters were determined for the MCP2 sample. The *C-t* curves were not smooth enough for MOC specimens to determine all the parameters, probably due to much lower cement paste content and coarse aggregate grains. This is in line with findings related to the *V_P_−t* curves where an increase in the aggregate content leads to less visible substages of the rapid *V_P_* increase period [2]. Therefore, only the initial setting was determined for the MOC specimens, as the time at the rapid drop of the *dC-t* curve. The final setting time was calculated considering the same setting periods of the MOM and MOC—which were equal to 90 min.

Initial and final setting times (Table 5) are in reasonably good agreement with values obtained by Staquet [56] for the same CBM compositions: 13.15 h and 14.82 h, respectively, in the case of the MOM, and 10.15 h and 11.43 h, respectively, in the case of the MOC. However, the setting times are much higher than in the case of ordinary compositions due to the retarding effect of the used superplasticizer. This is also true when the inflexion point PT2 (Figure 10) is considered. The inflexion point PT2 is a good approximation of the peak value on the calorimetry curve following the induction period. This peak value occurred at the age of 26 to 27 h for the MCP and MOM compositions and is often related to complete coverage of the C_3_S grains’ surface with the C-S-H “needles” and start of dense inner products formation [57]. At this stage, CBM is not capable of withstanding loads due to the specimen’s weight and handling operations. The PT2 time (26 to 27 h) also explains why during the de-moulding at 24 h, specimens disintegrated.

### 4.3. Hardened Properties of CBMs

The observation of hardened properties of CBMs began when specimens were able to withstand loads due to their weight and handling operations. For the studied compositions, this was only at the age of 30 h. In this chapter, the MCP, MOM and MOC specimens’ mechanical properties are presented at the specimens’ age of 30, 36, 48, 76 h, and 7 days. The equivalent age approach was not applied since the temperature of the specimens was not measured.

#### 4.3.1. Ultrasonic P and S Waves Transmission Velocity

**Figure 11:**
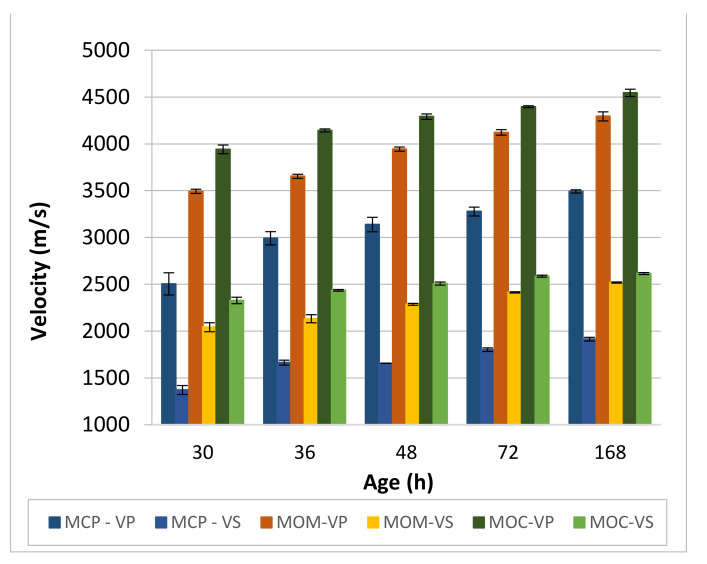
Development of transmission velocity of ultrasonic longitudinal (VP) and shear (VS) waves.

The ultrasonic P- and S-wave transmission time was measured for the three CBM compositions at each specimen’s age. The transmission path length for calculating the ultrasonic transmission velocity is 160 mm for MCP and MOM specimens and 400 mm for the MOC specimen. Figure 11 shows the development of the transmission velocity of ultrasonic P- and S-waves with the specimens’ age.

Shear waves are assumed inexistent in the CBM suspension after mixing since no shear forces are transmitted in fluids [2]. Thus, the S-waves can propagate in the CBM specimen only when enough cement hydration products are present to form an interconnected solid percolation path. This time is often defined as the initial setting time of specific CBM [2]. On the other hand, the velocity of P-waves at the initial setting time of cement paste is about 1450 m/s [58,59,60], which is the velocity of the P-waves in water. During the setting and hardening process of CBM, both velocities are continuously increasing and thus present promising parameters to monitor the solidification process.

By comparing velocities of the P-wave (*V_P_*) at the age of the specimens of 30 h (Figure 11) to results obtained on the same compositions by [55], it can be concluded that good reproducibility of the test results exists for the MCP and MOC compositions. Although an approach with the equivalent age and significantly smaller length of the test specimens were used in [55], measured average velocities were 2600 m/s and 4150 m/s for the MCP and MOC specimens, respectively. Values 2500 ± 120 m/s and 3940 ± 50 m/s were obtained for the MCP and MOC specimens, respectively, in our study. Based on the same *V_P_* measured on the MCP at 30 h, results from [55] can be used to estimate the *V_P_* at initial and final setting, and the PT2 time that presents roughly the start of the decelerated solidification stage of the MCP as 800 m/s, 1100 m/s and 2250 m/s, respectively. The *V_P_* values of initial and final setting agree with [61].

The P-wave velocity increase with the MCP specimens’ age is approximately constant (81 to 85 m/s per hour) between the PT2 time and 36 h. After that, the rate of the velocity increase started to decrease with time considerably. From these results, it can be concluded that the cement paste’s intense solidification process existed for up to 36 h, which was also confirmed by [55]. From 36 to 48 h, the increase in *V_P_* is highly reduced, and *V_S_* is lower at 48 h than 36 h. This implies the possible presence of cracks inside the MCP specimens, likely formed due to the cement paste’s autogenous shrinkage. The formation of cracks can break interconnected solid percolation paths for the S-waves. The ratio between the P- and S-wave velocities (*V_P_*/*V_S_*) is between 1.83 and 1.82 for 30, 72 and 168 h. However, it is reduced to 1.80 for 36 h and increased to 1.90 for 48 h. It seems that the change of the otherwise roughly constant *V_P_*/*V_S_* ratio of the MCP can be used as the measure to detect damages to the cement composite microstructure.

MOM specimens contain a larger number of solid fractions due to the incorporation of sand. Thus, higher values of the *V_P_* and *V_S_* were measured for these specimens (Figure 11). The *V_P_*/*V_S_* ratio is close to 1.71 for all but 48 h, where the ratio is increased to 1.73. This increase may also be due to crack formation, although the extent of the cracks seems to be much lower due to a smaller volume of cement paste and higher volume stability of the MOM compared to the MCP.

The highest *V_P_* and *V_S_* values were measured for the MOC due to the lowest cement paste content and the highest aggregate grains content, equal to 67%. The *V_P_*/*V_S_* ratio is close to 1.70 for all but 7 d (168 h), where the ratio is increased to 1.738. This value is close to the $\sqrt{3}$ which is the *V_P_*/*V_S_* ratio, often reported for stones [2].

#### 4.3.2. Dynamic and Static Elastic Properties of CBM Specimens

The simultaneous determination of the P- and S-ultrasonic wave transmission velocity makes it possible to determine the dynamic elastic parameters (*ν_d_, E_d_, G_d_*) of CBMs (Equations (1)–(3)). Those parameters are calculated for nondeformed specimens since very little force is applied to the specimen during the measurement. On the other hand, static elastic properties are determined on CBM specimens loaded to 25% to 40% of their strength—up to the load at which Hook’s law exists between stress and deformation. A compressive test is most often used to determine those parameters (Equations (6)–(8)).

**Figure 12:**
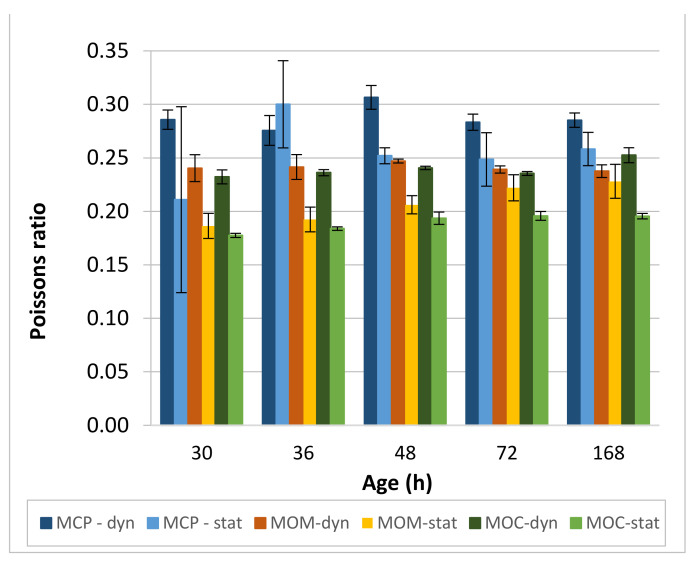
The comparison of dynamic and static Poisson’s ratio for CBMs.

The difference between static and dynamic Poisson’s ratio for studied CBMs is presented in Figure 12. For the MCP, *ν_d_* is close to 0.285 for 30, 72, and 168 h. Lower (0.276) and higher (0.307) values were measured at 36 and 48 h, respectively. Values of *ν_d_* below 0.30 are expected for the hydrated cement paste (HCP) since Poisson’s ratio of cement clinker minerals is 0.3 [62] and that of C-S-H outer and inner gel and of Ca(OH)_2_ is 0.25, 0.25, and 0.30, respectively [63]. The effect of formed cracks on the *V_P_* and *V_S_* values directly influenced calculated *ν_d_* at these two ages. The static Poisson’s ratio *ν_s_* is, as a rule, lower than the *ν_d_* and is around 0.25 at the age of the MCP 48 h or higher. On the other hand, at 30 and 36 h, the test results’ standard deviation was so high that the measured average value could not be applied as a realistic parameter. The presence of autogenous shrinkage cracks can be responsible for the observed behaviour. For MOM prisms, *ν_d_* is mostly in the range of 0.240 ± 0.002, except for the 0.247 ± 0.002 value at 48 h. Again, the presence of cracks in the specimens could influence the observed exception from the rule. The static Poisson’s ratios *ν_s_* of the MOM gradually increase, from 0.186 at 30 h to 0.228 at 168 h. A gradual increase of dynamic and static Poisson’s ratios with the specimen’s age was also observed for the MOC composition, where *ν_d_* increased from 0.232 at 30 h to 0.252 at 168 h, and *ν_s_* increased from 0.178 at 30 h to 0.196 at 168 h.

**Figure 13:**
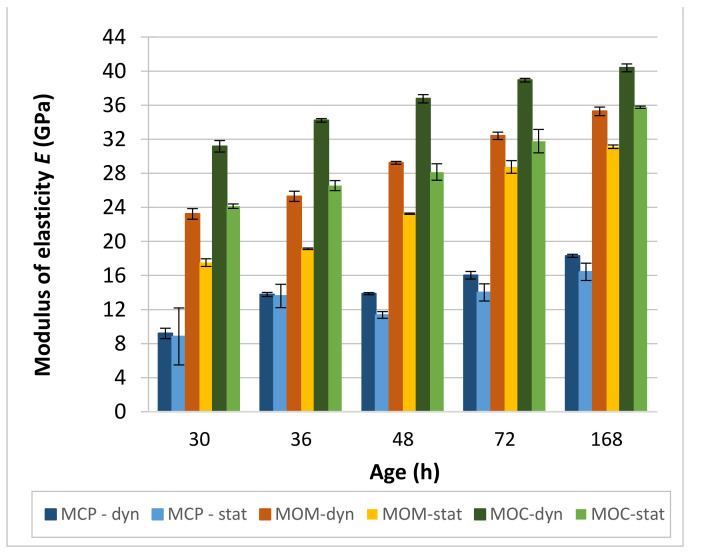
The comparison of dynamic and static modulus of elasticity (E-modulus) for CBMs.

The comparison between the dynamic and static E-moduli of studied CBMs is shown in Figure 13. In general, the elastic modulus is a function of densities of CBMs’ main components and the interface transition zone between the aggregate and cement matrix. It is common knowledge that the ordinary aggregate with a density between 2500 and 2700 kg/m^3^ has the most crucial influence on CBMs’ E-moduli and thus the E-moduli increase with the increase of volume fraction of aggregate grains; such aggregate was also used in this study. The dynamic elastic modulus (*E_d_*), determined by the non-destructive ultrasonic tests, is generally higher than the static elastic modulus (*E_s_*). When using the ultrasonic method, only negligible stress occurs in the specimen. Therefore, the specimen is not exposed to deformation and creep as it is during the static E-modulus test. Even so, creep deformations do not have a fundamental effect on the static elastic modulus, so the two methods are comparable [64]. The difference in dynamic and static E-moduli is mainly because CBMs’ behaviour is viscoelastic, which means that the behaviour of the material varies according to the strain rate applied during the test [65].

As expected, the lowest E-moduli were measured for the MCP composition, where there is no difference between *E_d_* and *E_s_* values for 30 and 36 h. At the age of 48 h, there is no increase in *E_d_*_,_ and *E_s_* is reduced compared to 36 h. Again, the possible presence of cracks can be responsible for the observed behaviour. There is another gradual increase in E-moduli at 72 and 168 h, and *E_d_* values seem to be slightly higher than that of *E_s_*. Comparison to the MCP’s E-moduli determined using the EMM-ARM test method that allows automatic and continuous evaluation of the property from final setting time forward [29] shows good agreement of the test results for all specimens’ ages. However, this is probably mainly because the two moduli are approximately the same for the MCP composition.

A significant difference was obtained between *E_d_* and *E_s_* moduli for the MOM compositions, with *E_d_* values notably higher (Figure 13). The ratio *E_d_*/*E_s_* is the highest at 30 and 36 h (1.32). With an age increase, it started to decrease, to 1.26 at 48 h and finally to 1.13 at 72 and 168 h. By comparing *E_d_* values with test results in [56], where the same MOM composition was tested, 30 h and 36 h values of 23.2 ± 0.6 GPa and 25.3 ± 0.6 GPa, respectively, are lower than 27.5 GPa and 28.3 GPa, respectively, reported by Staquet [56]. However, already at 48 h, the two *E_d_* are about the same (29.2 ± 0.2 GPa in our study and 30.5 GPa in [56]).

The ratio *E_d_*/*E_s_* for the MOC composition follows almost the same pattern as that observed at the MOM composition. The highest ratio of 1.3 was obtained at 30 h, 36 h and 48 h; at 72 h, a reduction to value 1.23 was observed. Finally, a ratio of 1.13 was obtained at 168 h. Due to the much higher volume and grain size of aggregate, the moduli of the concrete composition are considerably higher than those of the MOM. By comparing *E_d_* values with test results in [56], where the same MOC composition was tested, 30 h and 36 values of 31.2 ± 0.7 GPa and 34.2 ± 0.2 GPa in our study are only slightly lower than 33 GPa and 35 GPa, respectively, obtained by Staquet [56]. Again, at just 48 h, the two *E_d_* are about the same (36.8 ± 0.5 GPa in our study and 36.8 GPa in [56]). This also shows that the equivalent age approach would not influence the test results significantly, as this approach was applied in [56].

When comparing the *E_s_* results to the velocities of the shear waves (*V_S_*), the exponential relation of $E_{s}$=a⋅EXP(*b*⋅$V_{S}^{2}$), where a and b are constants, results in the highest coefficient of determination *R*^2^ equal to 0.83, 0.98 and 0.90 for the MCP, MOM and MOC, respectively. Moreover, by considering all test results (for MCP, MOM and MOC compositions), the correlation of $E_{s}=6.05\cdot EXP\left(0.255V_{S}^{2}\right)$ with *R*^2^ equal to 0.97 was obtained. Comparison of the *E_d_* and *E_s_* test results confirms the non-linear correlation between the two properties, as reported in [2]. By considering all test results, the correlation of $E_{s}=1.16\cdot E_{d}^{0.9}$ with *R*^2^ equal to 0.96 was obtained.

#### 4.3.3. Stress-Strain Diagrams of CBMs in Compression

**Figure 14:**
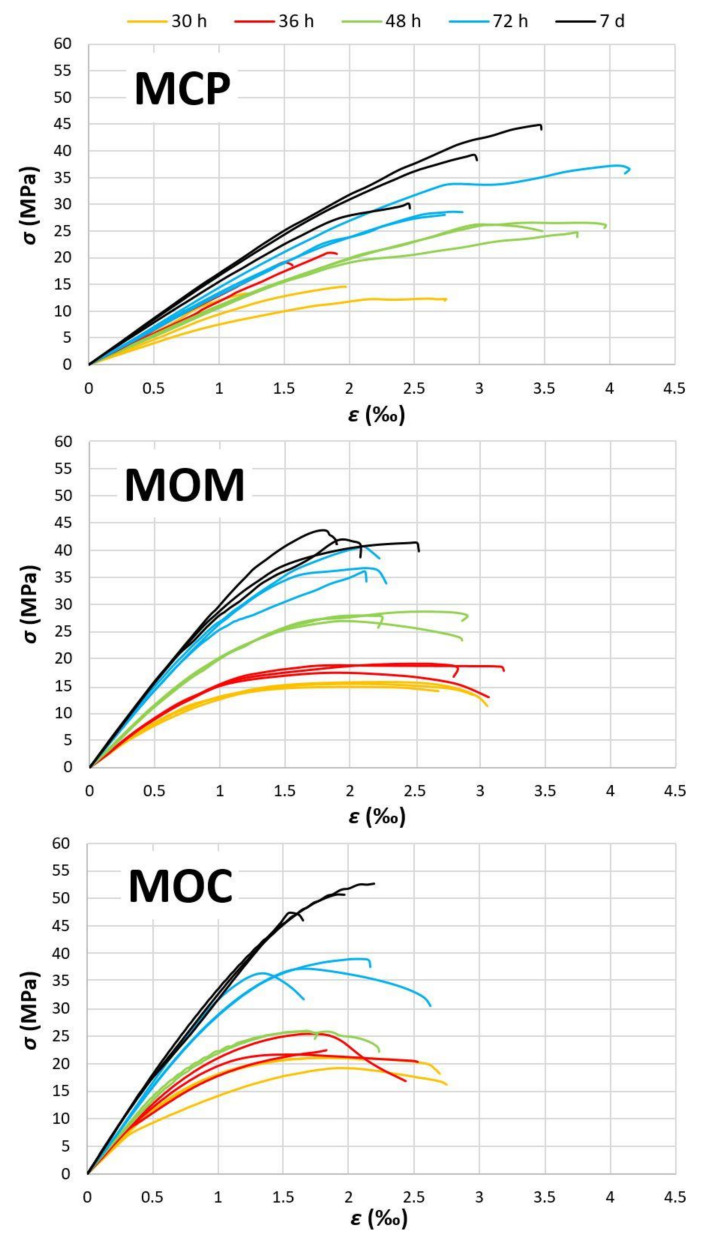
Stress-strain diagrams of CBM specimens after unloading elastic modulus test.

After the 3rd cycle of the unloading elastic modulus test, all the specimens were loaded to their total collapse. In this way, stress-strain diagrams (*σ*–*ε* behaviour) were obtained. For each tested CBM specimen, the obtained *σ*–*ε* diagram is shown in Figure 14. There are two exceptions where centrical loading was not possible—one MCP specimen at the age of 36 h and one MOC specimen at 48 h. These results were omitted from the analyses. The most dispersed *σ*–*ε* diagrams for particular age were observed for the MCP composition, as there is no aggregate in the mixture and the presence of cracks is plausible. These cracks can be present in the specimen before application of load or induced during the loading due to reduced tensile strength, both due to the autogenous shrinkage. It is also evident that at ages 48 h and higher, the MCP specimens possess higher deformability than the MOM specimens, although ultimate stresses are about the same for the particular age of specimens. A lower *E_s_* for the MCP specimens is responsible for the behaviour observed. Furthermore, MCP specimens show much more brittle behaviour than the MOM and MOC specimens, especially at ages 72 h and 168 h, where an almost linear behaviour of the MCP is observed. This is in line with the response shown in [66] for the HCP. As expected, the nonlinearity of *σ*–*ε* diagrams is increased when aggregate is introduced to the mixture. However, deformability is higher for the MOM specimens than the MOC specimens due to the lower modulus of elasticity.

The non-linear *σ*–*ε* behaviour is due to the gradual crack formation in the interface transition zone (ITZ) between the hardened HCP and aggregate grains. Usually, it is related to the lower strength of the ITZ due to the locally increased water-to-cement ratio as a consequence of vibration [66]. CBMs in fresh state possessed highly flowable consistency and no or minimum vibrations were needed to cast the specimens. Moreover, the MOC composition showed a brittle response at just 168 h, indicating that ITZ properties were similar to the bulk HCP ones. Due to the possibility of cracks formation, as a consequence of the autogenous shrinkage of the HCP, such cracks in the ITZ would have a similar influence on the *σ*–*ε* behaviour as the increased porosity ITZ. The cement binder’s self-healing capacity can eventually repair these cracks in the non-loaded specimen, resulting in a more brittle response of the MOC at 168 h.

#### 4.3.4. Strenght Properties of CBM Specimens

**Figure 15:**
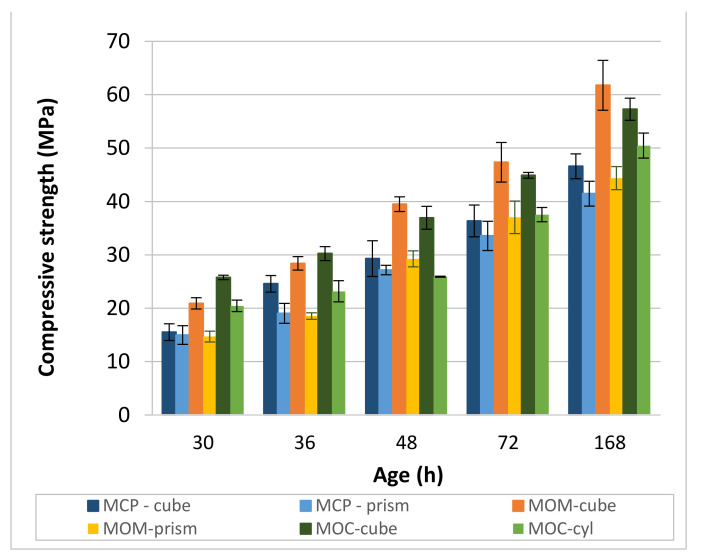
The influence of specimen’s shape on compressive strength for MCP, MOM, and MOC.

The influence of the specimen’s shape on compressive strength development is shown in Figure 15. As expected, higher strengths were measured on the cube specimens compared to prismatic or cylindrical 2/1 specimens. The shape conversion factor between the prism or cylinder and cube is not a constant value for a particular CBM. It seems to be dependent on the age of the specimen.

For the MCP specimens, the shape conversion factor is between 0.78 (at 36 h) and 0.97 (at 30 h) and is close to 0.9 at 48 h, 72 h and 168 h. This factor is much lower for the MOM composition, ranging between 0.65 (36 h) and 0.78 (72 h). The presence of sand influences the shape conversion factor. The lowest value of the factor was observed at 36 h for both CBM compositions. The presence of cracks that influence the strength of the prism more than the cube strength is a plausible cause for this observation. For the MOC, the shape factor is between 0.70 (48 h) and 0.88 (168 h), although the other three values are close to 0.8, which is the conversion factor usually used to calculate the cylinder (150/300 or 200/100 mm) compressive strength, from the 150 mm cube strength.

Direct comparison of the compressive strengths is possible only for the MCP and MOM compositions due to the same dimensions of specimens used. At 30 h and 36 h, the compressive strengths of the MCP and MOM were approximately the same when the 2/1 prism was used as a specimen, and the compressive strength is higher for the MOM composition when the cubic specimen was used. It seems that 2/1 prismatic specimens are a better choice when the compressive strength of the paste and mortar scale of the concrete is evaluated since they reflect the presence of cracks in the measured strength. Furthermore, one can conclude that MOM is not adversely affected by the aggregate incorporation, as reported in [66] since the compressive strength of the MOM is not lower compared to that of the MCP.

Comparing the MOC compressive strength to that obtained for the MOM composition is possible only if the shape conversion factor 1.12 is used to calculate the equivalent compressive strength of the 40 mm cube from the 150 mm cube. The comparison reveals that the MOC’s compressive strength is higher only for the 30 h and 36 h. At later ages, however, the compressive strengths of the two compositions are about the same. The standard cube (150 mm) compressive strength of the MOC reported in [55] is 30 MPa and 51 MPa for 48 h and 168 h, respectively. These values are slightly lower than 35 ± 2 MPa and 54 ± 2 MPa, respectively, obtained in our study.

When comparing the compressive strength results to the velocities of the shear waves (*V_S_*), the exponential relation of *f_C_* = a × EXP(b × *V_S_*), where a and b are constants, results in the highest coefficient of determination *R*^2^ equal to 0.95, 0.95 and 0.82 for the MCP, MOM and MOC, respectively.

**Figure 16:**
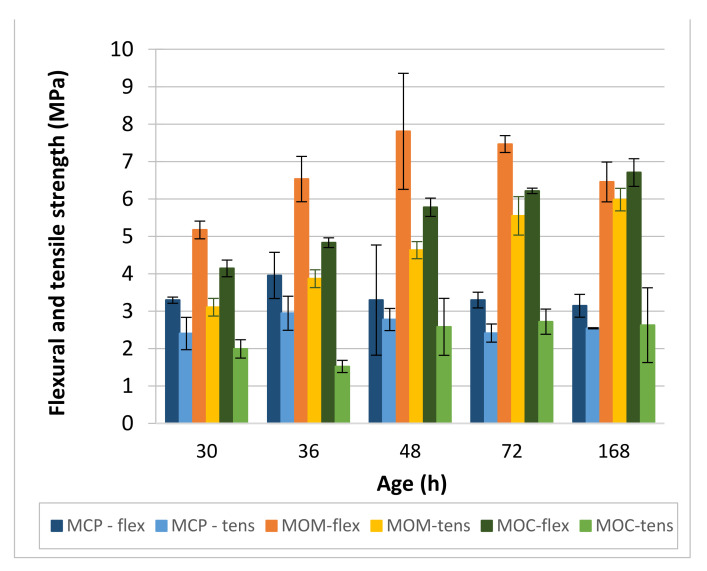
Flexural (flex) and splitting tensile (tens) strength of MCP, MOM, and MOC.

Flexural and splitting tensile strengths of the tested MCP, MOM and MOC mixtures are shown in Figure 16. Direct comparison is possible between the MCP and MOM compositions due to the identical prismatic specimens used. Tensile strength tests are much more sensitive to the presence of cracks compared to the compression test. For the MCP composition, tensile strength does not follow the compressive strength increase with the increase of the specimen’s age. Average values of flexural strength are between 3.14 MPa (168 h) and 3.96 MPa (36 h), and those of the splitting strength are between 2.40 MPa (30 h) and 2.94 MPa (36 h). Additionally, a high variation of the test results at 36 h and 48 h was observed. Observed behaviour is typical for specimens with induced cracks or other defects. Much higher flexural and splitting tensile strengths were obtained for the MOM specimens due to the incorporation of the fine aggregate. Average splitting strength values are gradually increasing with the specimens’ age, from 3.10 MPa (30 h) to 5.98 MPa (168 h). The situation is different for flexural strength, where the highest average value (7.81 MPa) and, at the same time, high standard deviation (1.55 MPa) were obtained at 48 h. Moreover, flexural strength at 168 h was lower compared to that at 72 h. From these results, one can conclude that the presence of cracks in MOM specimens is reflected in the change of flexural strength with the age of the specimen. For the MOC composition, flexural strength gradually increases with the specimens’ age, from 4.14 MPa at 30 h to 6.70 MPa at 168 h. The situation is different for the splitting strength, where the decrease of strength was observed from 30 h to 36 h (from average value 1.99 MPa to 1.52 MPa) and approximately the same average strength of 2.65 ± 0.07 MPa was maintained from 48 h to 168 h.

Observed behaviour is different from that reported in [55] for the same MOC composition, where splitting tensile strength was determined on 80/300 mm cylinders and a gradual increase of the specimen’s strength, from 1.1 MPa at 24 h to 2.2 MPa at 48 h and 3.5 MPa at 168 h, was reported.

As a rule, the development of the flexural and splitting tensile strength with the CBM age does not follow the compressive test pattern of a particular CBM. There is also a very poor correlation between the *V_S_* and flexural or splitting tensile strength of a particular CBM. There are only two exceptions—a good linear correlation was obtained at the MOM between *V_S_* and splitting tensile strength (*R*^2^ 0.92) and at the MOC between *V_S_* and flexural strength (*R*^2^ 0.95). It can be seen that no rule can be established between the MCP, MOM, and MOC properties. Several possible influencing parameters are responsible for the observed behaviour: random presence of cracks, non-adequate shape and dimensions of specimens to determine early age properties, and the possibility that elastic theory at the early age of CBMs is not valid for the destructive tests.

## 5. Conclusions

In this study, non-destructive and standard destructive tests were performed to evaluate cement-based material properties at three scales—the concrete, mortar, and paste scale. The research was carried out using materials and compositions proposed within the RRT^+^ programme of the COST Action TU1404. The studied compositions have increased autogenous shrinkage and a highly delayed setting, resulting in an extended early age period. The electrical conductivity measurements were used to follow a particular cement-based material solidification process, from the state of solid suspension up to the age of 7 days. On hardened specimens aged 30, 36, 48, 72 h, and 7 days, transmission times of ultrasonic P- and S-waves were measured simultaneously, and standard destructive tests were performed to determine both dynamic and static mechanical properties. Based on the experimental results obtained, the following main conclusions were drawn:

1. The electrical conductivity method used is capable of determining the initial and final setting time and the end of the solidification process acceleration stage (PT2) for the MCP and MOM compositions. For the MOC composition, only the initial setting time was determined. An increase in the aggregate content leads to less visible substages of the interval, where conductivity rapidly decreases with time.
2. Simultaneous P- and S-wave transmission velocities measurements revealed that the ratio *V_P_*/*V_S_* is highly dependent on the HCP and aggregate contents. The highest ratio of about 1.82 was obtained for the MCP. Lower ratios of 1.71 and 1.70 were obtained for the MOM and MOC, respectively. The only exception is MOC at 7 days, where a value of 1.738 was noted. The deviation from the otherwise roughly constant ratio *V_P_*/*V_S_* for each CBM may indicate cracks in the material at a particular age.
3. Comparison of dynamic and static elastic moduli, *E_d_* and *E_s_*, revealed almost no difference between the two moduli for the MCP. For the MOM and MOC compositions, the ratio *E_d_*/E_s_ was changed with the CBM’s age, from about 1.3 at 30 h to 1.13 at 168 h. Considering all test results (for MCP, MOM and MOC compositions), the non-linear correlation between the *E_d_* and *E_s_* was found to be in the form of $E_{s}=1.16\times E_{d}^{0.9}$ with *R*^2^ equal to 0.96. The *E_s_* results additionally correlate very well with the square of *V_S_*, as $E_{s}=6.05\times EXP\left(0.255V_{S}^{2}\right)$ with *R^2^* equal to 0.97.
4. Comparison of dynamic and static Poisson’s ratio, *ν_d_* and *ν_s_*, revealed that the value is approximately 0.285 for *ν_d_* and about 0.25 for *ν_s_* when MCP is considered. The *ν_d_* value between 0.24 and 0.25 was obtained for the MOM and MOC. Static Poisson’s ratio gradually increased with the specimen’s age. Values from 0.19 to 0.23 were obtained for the MOM, and from 0.18 to 0.20 for the MOC.
5. Results of compressive strength tests showed 32% higher MOM cube strengths compared to those of the MCP cubes. About the same cube compressive strength was measured for the MOM and MOC compositions, from 48 h up to 168 h. The cube compressive strength is highly correlated to the velocity of the S-waves (*V_S_*). However, a separate trendline exists for each CBM tested. This confirms that different parameters influence the compressive strength and static modulus of elasticity where the same $E_{s}$−$V_{S}^{2}$ tradeline exist for the three CBMs.
6. The comprehensive study using a combination of various non-destructive and destructive tests to evaluate the CBM at the three scales shows that evaluating the early age properties using commonly accepted destructive standard tests and specimens’ shape needs additional validation and possible modifications. Numerical simulations using reported test results can help solve existing issues.
7. Detection of cracks formed inside the CBM at an early age—such as autogenous shrinkage cracks—and possible self-healing of those cracks with the CBM’s age is of utmost importance. Loading the reinforced concrete elements when such cracks are present in the CBM’s microstructure may lead to the highly reduced service life or even the collapse of reinforced concrete infrastructure. A change in the otherwise roughly constant *V_P_*/*V_S_* ratio can indicate cracks in the CBM for the paste and mortar scale.

## Figures and Tables

**Table 1:** Chemical composition of cement.

Oxide (%)	SiO_2_	CaO	MgO	Al_2_O_3_	Fe_2_O_3_	SO_3_	Na_2_O	K_2_O	Cl
CEM I	21.3	63.8	1.9	3.5	4.3	2.7	0.09	0.59	0.05

**Table 2:** Composition of cement-based materials’ (CBMs) [33].

Basic Material	Type of the Material	MCP (kg/m^3^)	MOM (kg/m^3^)	MOC (kg/m^3^)
Cement	CEM I 52.5 N	1299	689	439
Dry sand	0–4 mm	\	1212	772
Fully saturated gravel	4–11 mm	\	\	525
	8–16 mm	\	\	424
Admixtures	Superplasticizer	5.85	5.85	3.73
Added water *	Tap water	514.8	280.1	178.4
w_eff_/c		0.4		

* Added water = Effective water (obtained from w_eff_/c ratio) − 0.8 * amount of the Sp (80% of Sp mass is water) + water theoretically absorbed by the sand (0.77% as a coefficient of absorption).

**Table 3:** Fresh properties of MCP and MOM.

Fresh Properties of CBM’s	Temperature of Mixture	Flow Value	Density
	(°C)	(mm)	(kg/m^3^)
MCP	19.9	309	1923
MOM	20.1	236	2254

**Table 4:** Fresh properties of MOC.

Mixture:	Temperature of Mixture	Air Content	Flow Value	Slump Value	Density
	(°C)	(%)	(mm)	(mm)	(kg/m^3^)
30 h	19.6	2.65	505	250	2370
36 h	18.5	2.7	535	250	2400
48 h	20.2	2.65	565	250	2390
72 h	19.9	2.6	525	254	2360
7 d	18.8	2.6	530	250	2380

**Table 5:** Characteristics time periods of the CBM solidification process.

Time Periods of the Hydration Process	MCP	MOM	MOC
Initial setting time (h)	13.2; 14.0	14.0	9.5
Final setting time (h)	14.8; 15.5	15.5	11.0
End of the solidification process acceleration stage—PT2 (h)	26; 26.5	26.5; 27	nd

## Data Availability

Data sharing is not applicable to this article.
